# Hearts deficient in both Mfn1 and Mfn2 are protected against acute myocardial infarction

**DOI:** 10.1038/cddis.2016.139

**Published:** 2016-05-26

**Authors:** A R Hall, N Burke, R K Dongworth, S B Kalkhoran, A Dyson, J M Vicencio, G W Dorn II, D M Yellon, D J Hausenloy

**Affiliations:** 1 The Hatter Cardiovascular Institute, University College London, London, UK; 2 Division of Medicine, University College London, London, UK; 3 Center for Pharmacogenomics, Department of Internal Medicine, Washington University School of Medicine, St Louis, MO, USA; 4 Cardiovascular and Metabolic Disorders Program, Duke-National University Singapore Graduate Medical School, Singapore, Singapore; 5 National Heart Research Institute Singapore, National Heart Centre Singapore, Singapore, Singapore

## Abstract

Mitochondria alter their shape by undergoing cycles of fusion and fission. Changes in mitochondrial morphology impact on the cellular response to stress, and their interactions with other organelles such as the sarcoplasmic reticulum (SR). Inhibiting mitochondrial fission can protect the heart against acute ischemia/reperfusion (I/R) injury. However, the role of the mitochondrial fusion proteins, Mfn1 and Mfn2, in the response of the adult heart to acute I/R injury is not clear, and is investigated in this study. To determine the effect of combined Mfn1/Mfn2 ablation on the susceptibility to acute myocardial I/R injury, cardiac-specific ablation of both Mfn1 and Mfn2 (DKO) was initiated in mice aged 4–6 weeks, leading to knockout of both these proteins in 8–10-week-old animals. This resulted in fragmented mitochondria (electron microscopy), decreased mitochondrial respiratory function (respirometry), and impaired myocardial contractile function (echocardiography). In DKO mice subjected to *in vivo* regional myocardial ischemia (30 min) followed by 24 h reperfusion, myocardial infarct size (IS, expressed as a % of the area-at-risk) was reduced by 46% compared with wild-type (WT) hearts. In addition, mitochondria from DKO animals had decreased MPTP opening susceptibility (assessed by Ca^2+^-induced mitochondrial swelling), compared with WT hearts. Mfn2 is a key mediator of mitochondrial/SR tethering, and accordingly, the loss of Mfn2 in DKO hearts reduced the number of interactions measured between these organelles (quantified by proximal ligation assay), attenuated mitochondrial calcium overload (Rhod2 confocal microscopy), and decreased reactive oxygen species production (DCF confocal microscopy) in response to acute I/R injury. No differences in isolated mitochondrial ROS emissions (Amplex Red) were detected in response to Ca^2+^ and Antimycin A, further implicating disruption of mitochondria/SR tethering as the protective mechanism. In summary, despite apparent mitochondrial dysfunction, hearts deficient in both Mfn1 and Mfn2 are protected against acute myocardial infarction due to impaired mitochondria/SR tethering.

New therapeutic strategies are required to protect the heart against acute ischemia/reperfusion (I/R) injury in patients with ischemic heart disease, the leading cause of death and disability worldwide. Acute myocardial I/R injury causes cardiomyocyte death by inducing mitochondrial Ca^2+^ overload, the production of mitochondrial oxidative stress, and the opening of the mitochondrial permeability transition pore (MPTP).^1, 2, 3^ Therefore, preventing these detrimental effects of acute myocardial I/R injury on mitochondrial function is an important therapeutic strategy for cardioprotection.

Mitochondria undergo continual cycles of fusion and fission, processes which are fundamental to mitochondrial health – a phenomenon that has been termed ‘mitochondrial dynamics'.^4, 5, 6, 7, 8^ Mitochondrial quality is maintained by mitochondrial fusion, which allows mixing of mitochondrial DNA, lipids, proteins, and metabolites, and mitochondrial fission, which allows the selective removal of damaged mitochondria by mitophagy.^9^ The key proteins mediating mitochondrial fusion are the Mitofusins (Mfn1 and Mfn2)^10^ and optic atrophy 1 (OPA1),^11^ while dynamin related protein 1 (Drp1)^12^ mediates mitochondrial fission by interacting with human fission protein (hFis1),^13^ mitochondrial fission factor (MFF),^14^ and mitochondrial dynamics proteins of 49 and 51 kDa (MiD49/MiD51).^15^ Mfn2 has a number of pleioptropic non-fusion effects including acting as a tether between mitochondria and the sarcoplasmic reticulum (SR).^16^

An optimal mitochondrial/SR tethering is essential for Ca^2+^ transfer from the SR to mitochondria and for maintaining cardiac bioenergetics. The dissociation of these organelles in murine hearts deficient in Mfn2 profoundly reduced Ca^2+^ transfer from the SR to the mitochondria. The uncoupling of mitochondrial ATP production from the energy requirements required by cardiac contraction^17^ has been shown to result in an overt hypertrophic phenotype, coupled with alterations in autophagy and ROS signaling.^18^

During I/R, mitochondria have been demonstrated to undergo Drp1-dependent fission in response to acute I/R injury. Accordingly, genetic or pharmacological inhibition of Drp1 has been reported to protect isolated cardiomyocytes against simulated I/R injury^19^ and reduce myocardial IS in both adult murine and rat hearts.^19, 20, 21, 22^ The role of the Mitofusins, Mfn1 and Mfn2, in the setting of acute myocardial I/R injury is not so clear. Therefore, in the current study, we investigated the acute effects of cardiac-specific ablation of both Mfn1 and Mfn2 on the susceptibility of the heart to acute I/R injury.

## Results

### Genetic knockout of Mfn1 and Mfn2

**Figure 1:**
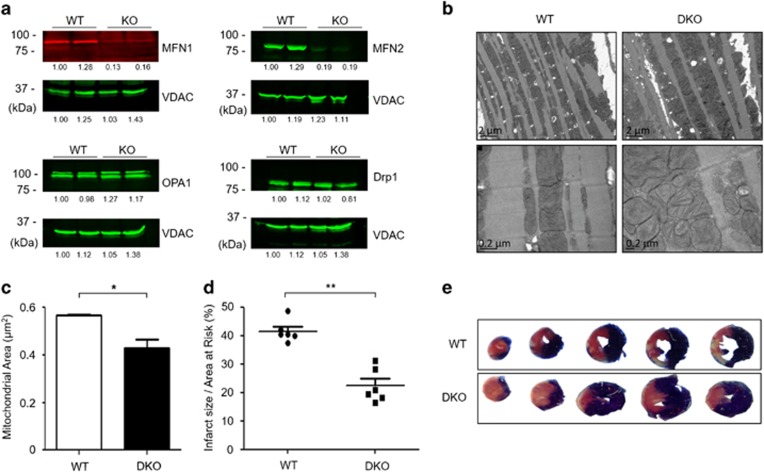
Acute ablation of both cardiac Mfn1 and Mfn2 altered mitochondrial morphology and protected the heart against acute myocardial infarction. (**a**) Western blotting confirmed the absence of both Mfn1 and Mfn2 in double knockout (DKO) hearts when compared with wild-type (WT) hearts with no significant difference in OPA1 or Drp1 protein levels (representative images). Representative quantifications are shown below the individual blots. (**b**) Electron microscopy shows changes in mitochondrial morphology in DKO hearts when compared with WT hearts, with fragmented reticular mitochondria and altered cristae structure (representative images – scale bars at 2 and 0.2 *μ*m, respectively). (**c**) Interfibrillar mitochondrial area is significantly reduced in DKO hearts compared with WT interfibillar mitochondria using 2D EM analysis. *N*=3 hearts/group with around 400 mitochondria analyzed per heart. Error bars indicate S.E.M. Nonparametric Mann–Whitney *t*-test. *P≤0.05. (**d**) Hearts deficient in both Mfn1 and Mfn2 (DKO) were resistant to acute I/R injury as evidenced by a significant reduction in myocardial IS when compared with WT hearts, expressed as IS as a percentage of the area-at-risk (AAR). *N*=6/group. Error bars indicate S.E.M. Unpaired *t*-test. **P≤0.001. (**e**) Representative images of short-axis heart slices following dual staining with Evan's blue and tetrazolium to stain the AAR and areas of infarction, respectively

Utilizing the MerCreMer inducible cardiac-specific knockout model, both Mfn1 and Mfn2 were genetically ablated in the adult murine cardiomyocytes by administration of tamoxifen in 4–6-week-old mice. Western blotting confirmed the ablation of both Mfn1 and Mfn2 between 8 and 10 weeks of age, the age at which all further experiments were conducted. There were no compensatory changes in protein expression of cardiac OPA1 or Drp1 in DKO compared with WT hearts (Figure 1a).

### Abnormal mitochondrial morphology in hearts deficient in both Mfn1 and Mfn2

Electron microscopy of hearts deficient in both Mfn1 and Mfn2 revealed predominantly fragmented interfibrillar mitochondria (smaller and reticular in shape) with loss of cristae structure when compared with WT hearts (Figures 1b and c).

### Hearts deficient in both Mfn1 and Mfn2 are protected against acute I/R injury

Hearts deficient in both Mfn1 and Mfn2 had no evidence of cardiomyopathy at 8–10 weeks, with normal left ventricular chamber dimensions and no evidence of left ventricular hypertrophy (normal heart weight/body ratio; Supplementary Figure 1a). Following *in vivo* acute myocardial I/R injury, the IS to area-at-risk ratio (IS/AAR%) was significantly reduced from 41±3.6% to 22±3.7% in DKO mice compared with WT littermates (Figures 1d and e). There was no difference in the size of the AAR (Supplementary Figure 1b).

### Impaired contractile function in hearts deficient in both Mfn1 and Mfn2

**Figure 2:**
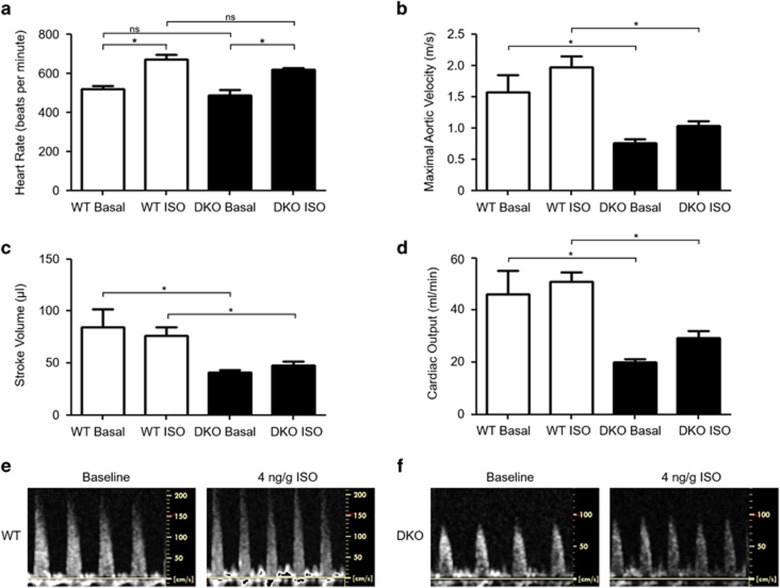
Knockout of Mfn1 and Mfn2 significantly reduced contractile power, stroke volume, and cardiac output without affecting heart rate. (**a**) Genetic knockout of Mfn1 and Mfn2 did not alter heart rate at baseline or in response to adrenergic stimulation. (**b**) Peak blood flow through the aorta is reduced in DKO mice at baseline and under isoproterenol (ISO) stimulation. (**c**) Cardiac stroke volume is reduced at baseline in DKO mice. (**d**) Cardiac output is reduced at baseline and under ISO stimulation in DKO mice. (**e**) Representative Doppler traces of blood flow in the aorta in WT mice at baseline and 4 min post ISO administration (4 ng/g body weight). (**f**) Representative Doppler traces of blood flow through the aorta in DKO mice at baseline and 4 min post ISO administration (4 ng/g body weight). *N*=3/group, and statistical analysis was performed by a one-way ANOVA with a Tukey post-test. *P≤0.05. Error bars indicate S.E.M.

DKO mice had evidence of impaired myocardial contractile function under basal conditions and in response to isoproterenol (ISO) stress with reduced aortic velocity (Figure 2b), reduced stroke volume (Figure 2c), and reduced cardiac output (Figure 2d) when compared with WT mice. However, heart rate (Figure 2a), fractional shortening, and both posterior and anterior wall thickness during both systole and diastole were unaffected when compared with WT hearts (Supplementary Figures 2a–e).

### Impaired mitochondrial respiratory capacity in hearts deficient in both Mfn1 and Mfn2

**Figure 3:**
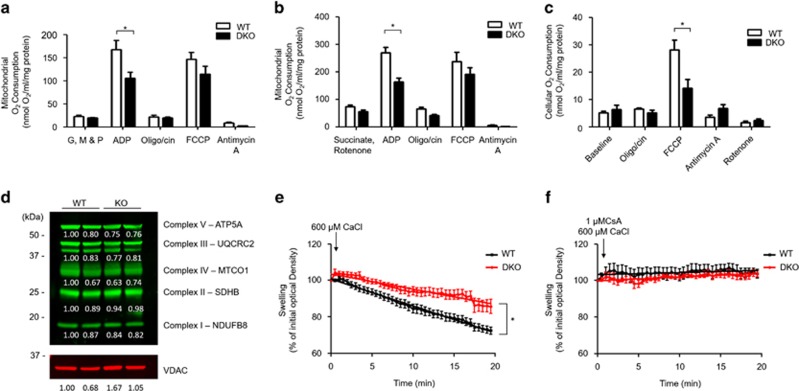
Acute ablation of both cardiac Mfn1 and Mfn2 resulted in impaired mitochondrial respiratory function. (**a**) ADP-stimulated mitochondrial respiration in the presence of complex I substrates (GMP – Glutamate, Malate, and Pyruvate) was impaired in DKO cardiac mitochondria compared with WT cardiac mitochondria. (**b**) ADP-stimulated mitochondrial respiration in the presence of complex II substrate (Succinate) and the complex I inhibitor Rotenone was impaired in DKO cardiac mitochondria compared with WT cardiac mitochondria. (**c**) The maximal oxygen consumption rate (due to uncoupling by FCCP) was decreased in isolated DKO ventricular cardiomyocytes compared with WTs. *N*=5 respiratory run per group, mitochondria were pooled from 15 mice of each genotype, and statistical analysis was performed by a two-way ANOVA. *P<0.05. Error bars indicate S.E.M. (**d**) Expression of mitochondrial subunits is not significantly altered by dual KO of Mfn1 and Mfn2 at the timepoint studied. (**e**) Mitochondrial swelling induced by 600 *μ*M CaCl_2_ is inhibited in DKO mitochondria when compared with WT mitochondria. (**f**) Mitochondrial swelling induced by 600 *μ*M CaCl_2_ is inhibited the MPTP inhibitor cyclosporin-A (CsA, 1 *μ*M). *N*=3 triplicate runs of mitochondria isolated from nine mice. Error bars indicate S.E.M. and statistical analysis was performed by analysis of the linear regression. *P<0.05

To test whether Mfn1/Mfn2 deficiency impacts upon mitochondrial oxygen consumption, respiratory function was measured in both isolated mitochondria and isolated intact cardiac ventricular cardiomyocytes. ADP-stimulated mitochondrial respiration (state 3) was significantly impaired in mitochondria isolated from DKO cardiac tissue, when the functionality of complex I and complex II was tested, compared with WT mitochondria (Figures 3a and b). A trend towards a reduced maximal respiration (FCCP stimulated) was observed in DKO compared with WT mitochondria (Figures 3a and b). In addition, a significant reduction in maximal respiration was observed in DKO intact cardiomyocytes compared with WT (Figure 3c), although this was not associated with a reduction in expression of the respiratory complexes (Figure 3d).

### Resistance to MPTP opening in cardiomyocytes deficient in both Mfn1 and Mfn2

DKO cardiac mitochondria were also found to be resistant to MPTP opening as evidenced by less mitochondrial swelling in response to Ca^2+^ when compared with WT mitochondria (Figure 3e). Ca^2+^-induced mitochondrial swelling was prevented by the known MPTP inhibitor, CsA, confirming that this experimental model was assessing MPTP opening (Figure 3f).

### Less mitochondrial Ca^2+^ overload during simulated I/R in cardiomyocytes deficient in both Mfn1 and Mfn2 due to a reduced mitochondria–SR interaction

**Figure 4:**
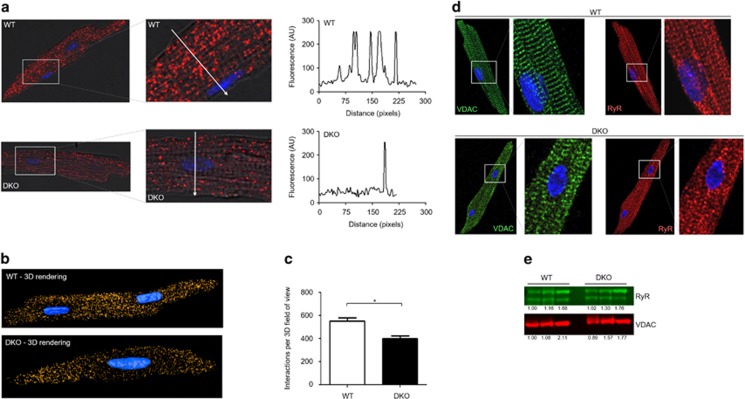
Acute ablation of both cardiac Mfn1 and Mfn2 reduces the interaction between the mitochondria and SR. (**a**) 2D confocal images showing the points of interaction between mitochondria and the SR in myocytes isolated from WT and DKO hearts, as visualized by the red dots, the nucleus is visualized in blue. Magnified insets show the zone of a line-scan fluorescence profile analysis (white arrow), plotted in the right. (**b**) Whole-cell 3D rendering from multiple z-stack images, compiling the points of interaction between the mitochondria and the SR in myocytes isolated from WT and DKO hearts, as visualized in gold, with the nucleus visualized in blue. (**c**) Quantification of the whole-cell 3D interactions visualized between the mitochondria and the SR. Antibodies to VDAC and the ryanodine receptor (Pan-RyR) were used as to mark the mitochondria and SR, respectively, prior to the proximal ligation assay. *N*=80 cells from three heart isolations. Error bars indicate S.E.M. and statistical analysis was performed by an unpaired *t*-test. *P≤0.05. (**d**) Representative immunocytochemistry images of myocytes isolated from WT and DKO hearts stained with antibodies for VDAC (green) and Pan-RyR (red). (**e**) Representative western blot image of VDAC and RyR expression in whole heart homogenate taken from WT and DKO mice

**Figure 5:**
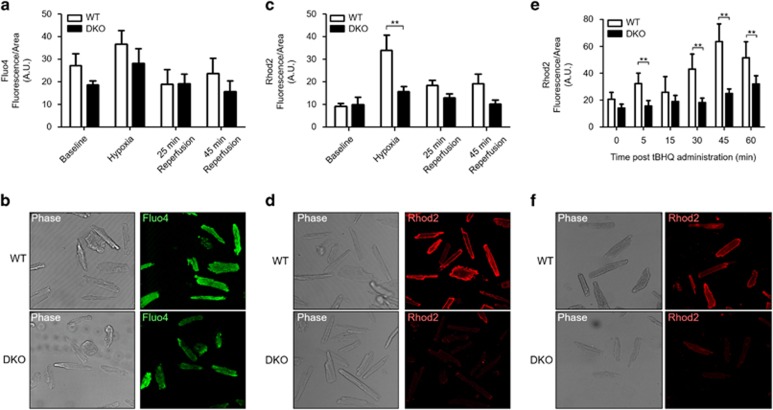
Acute ablation of both cardiac Mfn1 and Mfn2 attenuated mitochondrial Ca^2+^ uptake during acute I/R injury. (**a**) There was no significant difference in cytosolic Ca^2+^ (measured by Fluo4 fluorescence) between the genotypes under baseline conditions, at the end of simulated ischemia and 25 and 45 min after simulated reperfusion, between DKO and WT ventricular cardiomyocytes (two-way ANOVA *P*>0.05, comparing genotypes across the I/R treatment). (**b**) Representative phase contrast and confocal Fluo4 images of WT and DKO cardiomyocytes taken at the end of simulated ischemia. (**c**) Mitochondrial Ca^2+^ handling during simulated ischemia-reperfusion was significantly different between the two genotypes (two-way ANOVA between genotypes *P*=0.002). Bonferroni post-test revealed a significant increase in WT mitochondrial Ca^2+^ during ischemia (*P*>0.01), with no differences in mitochondrial Ca^2+^ under baseline conditions, and 25 and 45 min after simulated reperfusion, between DKO and WT ventricular cardiomyocytes. (**d**) Representative phase contrast and confocal Rhod2 images of WT and DKO cardiomyocytes taken at the end of simulated ischemia. (**e**) Treatment with 50 *μ*M tBHQ (SERCA inhibitor) stimulated a gradual increase in mitochondrial Ca^2+^ (measured by Rhod2 fluorescence) in WT cardiomyocytes, which peaked 45 min after initial administration. In DKO myocytes, the increase in Rhod2 fluorescence stimulated by tBHQ was delayed, with a marginal increase after 60 min. A two-way ANOVA revealed a significant difference in mitochondrial Ca^2+^ handling between the genotypes across the time points studied (*P*=0.0004). (**f**) Representative phase contrast and confocal Rhod2 images of WT and DKO cardiomyocytes taken after 45 min of tBHQ treatment. *N*=4 mice/group. Error bars indicate S.E.M. and statistical analysis was performed by a two-way ANOVA with a Bonferroni post-test. **P≤0.001

With Mfn2 reported to mediate tethering between the mitochondria and SR, we hypothesized that Ca^2+^ transfer from the SR to mitochondria would be decreased in DKO hearts and so there may be a difference in mitochondrial Ca^2+^ handling during acute I/R injury. First, we assessed the interaction between the mitochondria and the SR in ventricular cardiomyocytes isolated from WT and DKO using the proximal ligation assay (interaction detection range 0–40 nm), targeting the SR Ca^2+^ channel RyR and the mitochondrial Ca^2+^ channel VDAC. The degree of interaction was significantly reduced in DKO cardiomyocytes when compared with WT cardiomyocytes, using both two-dimensional and three-dimensional analyses (Figures 4a and c). To control that the decreased interaction between RyR and VDAC observed in the PLA data was not an artifact due to a different expression of these proteins in the DKO model, we performed conventional immunocytochemistry experiments and western blotting. We validated that their expression was comparable in WT and DKO cells for both biochemical techniques (Figures 4d and e), and importantly, that their subcellular distribution was also maintained in the DKO cells. This validates the PLA result, confirming that despite being present in a striated pattern, which may suggest close physical localization, both the RyR and VDAC require a molecular micro-domain to interact, in which Mfn proteins are essential mediators. We observed no significant differences in cytosolic Ca^2+^ between WT and DKO cardiomyocytes at baseline or in response to acute I/R injury to explain the observed differences in mitochondrial Ca^2+^ loading (Figures 5a and b), suggesting unaltered cytosolic Ca^2+^ handling in DKO hearts. However, there was a significant increase in mitochondrial Ca^2+^ levels during simulated ischemia in WT cardiomyocytes, which was absent in DKO cells (Figures 5c and d). To confirm the differences in mitochondrial Ca^2+^ transfer from the SR observed between WT and DKO cells results, we administered the SERCA inhibitor tert-butylhydroquinone (50*μ*M, tBHQ). In WT myocytes, a steady mitochondrial Ca^2+^ uptake was observed as an increase in Rhod2 fluorescence, which was delayed in DKO cells (Figures 5e and f). Together, these results suggest an impaired mitochondrial Ca^2+^ uptake in the DKO hearts.

### Less oxidative stress production in cardiomyocytes deficient in both Mfn1 and Mfn2

**Figure 6:**
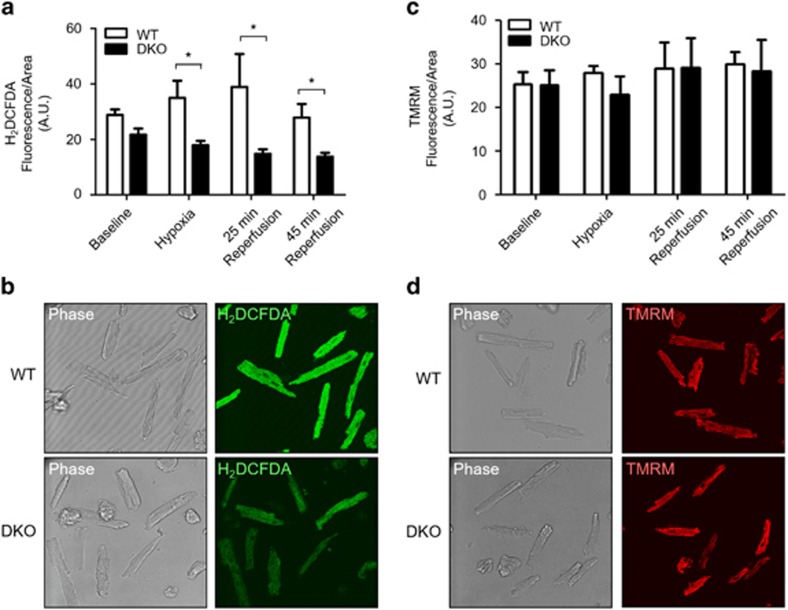
Acute ablation of both cardiac Mfn1 and Mfn2 attenuated production of oxidative stress during acute I/R injury. (**a**) The increase in oxidative stress (measured by H_2_DCFDA fluorescence) observed in WT cardiomyocytes during simulated ischemia and reperfusion was attenuated in DKO cardiomyocytes. There was a statistical difference in oxidative stress handling between the two genotypes across the I/R protocol (*P*<0.001, two-way ANOVA), with further statistical significance at 25 min after simulated reperfusion (Bonferroni post-test *P*<0.01). (**b**) Representative phase contrast and confocal H_2_DCFDA images of WT and DKO cardiomyocytes taken at the end of simulated ischemia. (**c**) There was no difference in mitochondrial membrane potential (measured by 30 nM TMRM fluorescence) under baseline conditions, at the end of simulated ischemia and 25 and 45 min after simulated reperfusion, between DKO and WT ventricular cardiomyocytes (two-way ANOVA with Bonferroni post-test). (**d**) Representative phase contrast and confocal and TMRM images of WT and DKO cardiomyocytes taken at the end of simulated ischemia. *N*=4 mice/group. Error bars are S.E.M and statistical analysis was performed by a two-way ANOVA. *P≤0.05

**Figure 7:**
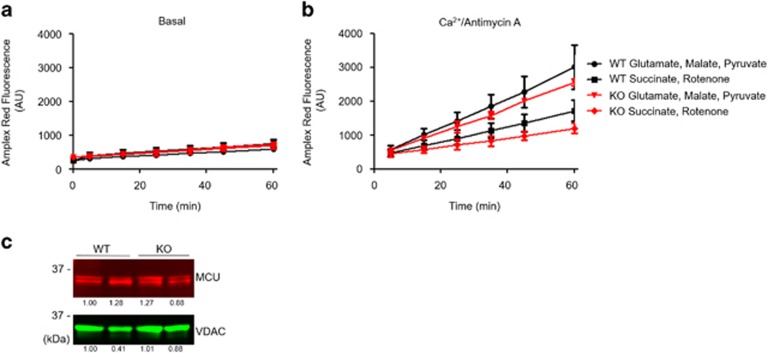
Acute ablation of both cardiac Mfn1 and Mfn2 does not alter mitochondrial H_2_O_2_ emission. (**a**) Mitochondrial production of H_2_O_2_ was measured by Amplex Red. Production of reactive oxygen species is similar between WT and DKO isolated mitochondrial at baseline. *N*=3 triplicate runs of mitochondria isolated from nine mice. (**b**) Mitochondrial production of H_2_O_2_ in the presence of 100 *μ*M Ca^2+^ and 1 *μ*g/ml Antimycin A (to mimic I/R) was similar between WT and DKO isolated mitochondria. *N*=3 triplicate runs of mitochondria isolated from nine mice. (**c**) Expression of the mitochondrial Ca^2+^ uniporter is unaffected by KO of the Mfn proteins

No differences in oxidative stress between WT and DKO cardiomyocytes were observed under baseline conditions. However, in response to simulated ischemia, there was an increase in oxidative stress in the WT cardiomyocytes, which was attenuated in DKO cells (Figures 6a and b). During simulated reperfusion, there was a similar difference in oxidative stress, with DKO demonstrating a significant reduction compared with WT cardiomyocytes. Importantly, the differences in mitochondrial Ca^2+^ or ROS levels were not due to changes in mitochondrial membrane potential as this did not differ between WT and DKO cells (Figures 6c and d). To assess whether the differences in ischemic oxidative stress were mediated by changes in Ca^2+^ transfer due to mitochondria–SR tethering, rather than due to Ca^2+^ uptake defects intrinsic to mitochondria in the DKO hearts, H_2_O_2_ emissions were measured in mitochondria isolated from WT and DKO hearts. The addition of Ca^2+^ and Antimycin A (to simulate ischemic Ca^2+^ overload and electron transfer chain inhibition) significantly increased H_2_O_2_ production compared with baseline, although to the same extent in WT and DKO mitochondria (Figures 7a and b). No changes in mitochondrial calcium uniporter expression (Figure 7c) (the principal pathway through which Ca^2+^ enters the mitochondria) were observed. These data imply that the reduced ROS generation observed in isolated DKO myocytes (Figure 6c) was because of a reduced Ca^2+^ overload resulting from the disassociation of the mitochondria from the SR, and not to intrinsic Ca^2+^ uptake deficiencies in DKO mitochondria.

## Discussion

We found that the acute ablation of both cardiac Mfn1 and Mfn2 protected the heart against acute myocardial infarction. This was despite abnormal mitochondrial morphology, decreased mitochondrial respiratory function, and impaired myocardial contractile function. As well as an intrinsic resistance to MPTP opening, the cardioprotection was associated with an attenuated mitochondrial Ca^2+^ overload and oxidative stress in DKO cardiomyocytes subjected to acute I/R injury as a result of a disrupted mitochondria–SR tethering.

Although genetic or pharmacological inhibition of Drp1 has been shown to inhibit ischemia-induced mitochondrial fission and protect the heart against acute I/R injury,^19, 20, 21, 22^ the role of the Mitofusins in this setting is not so clear. *In vitro*, using the HL-1 cardiac cell line, it has been shown that the overexpression of either Mfn1 or Mfn2 attenuated cell death induced by simulated I/R injury.^19^ In contrast, the siRNA knockdown of Mfn2 worsened cell survival following simulated I/R injury in neonatal rat cardiomyocytes.^23^ These findings suggest that the Mitofusins may have a cardioprotective role in cellular models of acute I/R injury. However, in the adult heart, the cardiac-specific ablation of either Mfn1 or Mfn2 have produced unexpected and contrasting results in terms of cellular protection. Adult murine cardiomyocytes deficient in Mfn1 have been shown to be resistant to both MPTP opening and cell death induced by oxidative stress,^24^ and cardiomyocytes isolated from Mfn2-deficient adult mice were found to be resistant to MPTP opening and were protected against simulated I/R injury.^23^ The reason for these discordant findings is not clear, but it may relate to the pleiotropic actions of the Mitofusin proteins.^25^ In this regard, Mfn2 has been shown to be required to tether SR to mitochondria,^16^ which promotes efficient Ca^2+^ transfer to sustain bioenergetic demands. However, Mfn2 is also essential for mitochondrial fusion, and its absence promotes a fragmented mitochondrial network, which would be intrinsically less resistant to I/R injury. Other reported functions of Mfn2 are to mediate autophagy by facilitating the fusion of autophagosomes with lysosomes,^26^ and its absence promotes accumulation of damaged mitochondria, which are also more susceptible to damage. On the basis of these different functions of Mfn2, the question arises as to which of these functions is more important in an acute setting of damage such as I/R injury.

In our study, we investigated the acute effect of ablating the Mitofusins on the susceptibility of the heart to acute I/R injury. We found that hearts deficient in both Mfn1 and Mfn2 were resistant to acute myocardial infarction, and that this cardioprotective phenotype was due to beneficial effects on mitochondrial Ca^2+^ levels, oxidative stress, and MPTP opening – key mediators of cardiomyocyte death in the setting of acute I/R injury. Our data suggested that acute ablation of cardiac Mfn1 and Mfn2 attenuated mitochondrial Ca^2+^ accumulation and decreased oxidative stress production during acute I/R injury. Despite the hypothetical susceptibility of fragmented mitochondria to stress, our findings in which we observed decreased ER-mitochondrial contact demonstrate that this function is critical for the protection against I/R injury in the DKO hearts.

Mitochondrial uptake of Ca^2+^ via the mitochondrial calcium uniporter from the SR in response to acute I/R injury induces MPTP opening at the onset of reperfusion and is a major determinant of cardiomyocyte death.^27^ Preventing mitochondrial Ca^2+^ overload during acute I/R injury has been shown to attenuate cardiomyocyte death and reduce myocardial IS.^28, 29, 30^ In our study, we found that the increase in mitochondrial uptake of Ca^2+^ induced by acute I/R injury was absent in hearts deficient in both Mfn1 and Mfn2 and the potential mechanism for this is discussed in the following section.

Owing to the low affinity of the mitochondrial calcium uniporter for Ca^2+^, the transfer of Ca^2+^ from the SR to mitochondria requires these two organelles to be closely juxtaposed, as this allows the formation of subcellular micro-domains of high Ca^2+^ concentration.^16^,^17^ This spatial relationship between SR and mitochondria is required to couple the energy demands of excitation–contraction with mitochondrial ATP production. In this regard, Mfn2 has been shown to act as a tether between the SR and mitochondria, and hearts deficient in Mfn2 have been demonstrated to develop a chronic cardiomyopathy.^17^ Here, we confirm these results, demonstrating a reduced interaction between the mitochondria and the SR through the proximal ligation assay. The effect of this was a reduced mitochondrial Ca^2+^ overload observed during simulated I/R. The observations of no differences in cytosolic Ca^2+^ handling between WT and DKO cardiomyocytes but a reduced mitochondrial Ca^2+^ uptake in DKO cells during hypoxia is consistent with inefficient tethering of mitochondria to SR in the absence of Mfns. We propose that the acute effects of ablating both cardiac Mfn1 and Mfn2 would be to uncouple the SR from mitochondria, thereby reducing the mitochondrial uptake of Ca^2+^ and preventing mitochondrial Ca^2+^ overload during acute I/R injury. The reduced mitochondrial Ca^2+^ levels could also correlate with the reduced cardiac output observed in DKO mice when assessed by echocardiography, as a result of impaired bioenergetics.

We also observed reduced endogenous mitochondrial respiratory function in the DKO mice, at both complex I and complex II. Inhibition of mitochondrial respiration is well documented to be protective in the setting of I/R through a reduced oxygen consumption, and reduced ROS production.^31, 32, 33, 34, 35^ Therefore, there appears to be two mechanisms that converge to reduce mitochondrial ROS production in DKO cardiomyocytes during I/R, a reduced mitochondrial Ca^2+^ overload and a reduced mitochondrial respiratory capacity. We hypothesize that the 4 weeks of decreased Ca^2+^ transfer from the SR to mitochondria contributed to the decrease in respiratory capacity that we observed in the DKO mice.

Oxidative stress produced in response to acute I/R injury is a critical inducer of MPTP opening and cardiomyocyte death at the onset of myocardial reperfusion. Preventing the production of oxidative stress during acute I/R injury has been shown to inhibit MPTP opening, attenuate cardiomyocyte death, and reduce myocardial IS.^36^ In our study, we found that H_2_O_2_ production from isolated mitochondria was no different between genotypes, however, in isolated myocytes, the increase in oxidative stress induced by acute I/R injury was attenuated in hearts deficient in both Mfn1 and Mfn2. In these hearts, the reduction in mitochondrial Ca^2+^ uptake would be expected to impair mitochondrial respiratory function, the results of which would be decreased production of oxidative stress. Clearly, impaired mitochondrial respiratory function would be expected to be detrimental in the long-term and accounts for the cardiomyopathy observed in hearts lacking Mitofusins^37^ – however, in the short term, it may be beneficial in the response to acute I/R injury. It has been previously documented that transient impairment of mitochondrial respiration can protect the heart against acute I/R injury by attenuating the production of oxidative stress.^38^

The opening of the MPTP in the first few minutes of reperfusion uncouples mitochondrial oxidative phosphorylation resulting in ATP depletion and cardiomyocyte death, and preventing MPTP at this time has been reported to attenuate cell death and reduce myocardial IS. In our study, we found that MPTP opening susceptibility was decreased in the DKO cardiac mitochondria. Interestingly, cardiomyocytes with individual knockouts of Mfn1 and Mfn2 have been reported to be resistant to stress-induced MPTP opening.^23, 24^ Whether this is a direct or indirect effect upon the formation of the MPTP is unknown, but is likely to contribute to the protective phenotype observed here.

In summary, we have shown that acute ablation of both cardiac Mfn1 and Mfn2 rendered the heart resistant to acute myocardial infarction. We found that disruption to the interactions between the mitochondria and SR have an important role in reducing mitochondrial Ca^2+^ overload, attenuating oxidative stress, while dual KO of Mfn1 and Mfn2 inhibit MPTP opening, contributing to this cardioprotective phenotype. Of interest, the non-fusion pleiotropic effect of the Mitofusins in tethering the SR to mitochondria appeared to be more important for cellular protection against acute I/R injury than changes in mitochondrial morphology *per se*. It is important to note that although the acute ablation of Mfn1 and Mfn2 appeared to be protective against acute I/R injury, the long-term effects of ablating these proteins would not be beneficial and has been shown to result in a cardiomyopathy and sudden cardiac death.^37^ Therefore, we would propose transient inhibition of the Mitofusins during acute I/R injury to be a novel strategy for cardioprotection.

## Materials and Methods

All animal experiments were conducted in accordance with the Animals (Scientific Procedures) Act 1986 published by the UK Home Office and the Guide for the Care and Use of Laboratory Animals published by the US National Institutes of Health 1996. All laboratory reagents were purchased from Sigma, Poole, UK, unless otherwise stated. A more detailed methods section is found in the Supplementary Methods section.

### Genetic mice, myocardial infarction and cardiac phenotyping

Cardiomyocyte-specific ablation of both Mfn1 and Mfn2 (DKO) was initiated in mice aged 4–6 weeks using 5 days i.p. administration of tamoxifen (20 mg/kg/day) (*Myh6*-MerCreMer inducible model).^39^ WT control mice were flx/flx littermates injected with tamoxifen, but lacking the Cre-recombinase gene. All experiments were performed in mice aged 8–10 weeks. The acute effects of cardiac-specific ablation of both Mfn1 and Mfn2 on the susceptibility of acute I/R injury was investigated using an *in vivo* model of acute myocardial infarction as described previously.^40^ AAR and myocardial IS were determined and results presented as IS/AAR%. Cardiac phenotyping was performed by transthoracic two-dimensional echocardiography with Vivid 7 Dimension and 14 MHz probe (GE Healthcare, Hatfield, UK) as previously described^40^ at baseline and 4 min following ISO challenge (4 ng/g of body weight by intraperitoneal bolus).

### Western blotting and electron microscopy

Isolated cardiac myocytes were analyzed using standard western blotting techniques with the following antibodies (Abcam, Cambridge, UK; unless stated otherwise) for Mfn1 (ab57602), Mfn2 (ab124773), OPA1 (ab90857), Drp1 (#8570 Cell Signalling, Hitchin, UK), OxPhos antibody cocktail (ab110413) and VDAC (ab15895). The acute effects of cardiac-specific ablation of both Mfn1 and Mfn2 on mitochondrial morphology were investigated using electron microscopy, as previously described.^19^ Mitochondrial area was analyzed using ImageJ to tracing the outer mitochondrial membrane in 2D EM images. Mitochondrial area was calculated accordingly.

### Isolated cardiomyocyte studies

Ventricular cardiomyocytes were isolated from WT and DKO hearts using Liberase digestion as described previously.^41^ Mitochondrial respiratory function was assessed in WT and DKO cardiomyocytes using an Oxytherm (Hansatech, King's Lynn, UK) in an adapted protocol.^42^ Cytosolic and mitochondrial Ca^2+^, oxidative stress, and mitochondrial membrane potential were measured under basal conditions and in response to acute I/R injury in WT and DKO cardiomyocytes using Fluo4 (for cytosolic calcium), CM-H_2_DCFDA (for oxidative stress), and TMRM (for mitochondrial membrane potential). Cells were loaded with Rhod-2 and imaged prior to and during treatment with 50*μ*M tert-butylhydroquinone (tBHQ). For the PLA assay, isolated myocytes were fixed in 4% PFA, permeabilized in PBS with 0.3% triton x100 for 15 min on ice prior to blocking for 1 h at RT with PBS 5% BSA. Primary antibodies for VDAC (#4661, Cell Signalling) and the RyR (Pan-RyR, #MA3-925, Thermo Scientific, Paisley, UK) were incubated overnight (1:500). After washes in PBS, the proximal ligation assay (Sigma, UK) was performed according to the manufacturer's instructions. All images were acquired using a Leica confocal microscope (Leica, Wetzlar, Germany), ImageJ was used to assess fluorescence intensity (Fluo4, Rhod2, DCF, and TMRM) and number of PLA interactions. Regions of interest were traced using the phase image, and ROIs transferred to the fluorescent channel, where channel intensity was measured, and normalized to cell area.

### Isolated mitochondrial studies

Cardiac mitochondria were isolated from WT and DKO mice as described previously^43^ through differential centrifugation. Mitochondrial respiratory function was measured using an Oxytherm (Hansatech) as previously described.^44, 45^ Mitochondrial H_2_O_2_ emission was measured using Amplex Red (Invitrogen, Paisley, UK) in isolated mitochondria (500 *μ*g/ml) suspended in Miro5 respiration buffer, according to the manufacturer's instructions. Complex I activity was stimulated by the addition of 5 mM Glutamate, 5 mM Malate, and 1 mM Pyruvate, and complex II activity by the addition of 10 mM Succinate and 0.5 mM rotenone. Susceptibility to MPTP opening was investigated in isolated cardiac mitochondria using a calcium-induced mitochondrial swelling assay, as previously described^45^ with optical density measured by spectrophotometry (FLUOstar Omega, BMG Labtech, Cary, NC, USA) for 20 min. Treatment with the known MPTP inhibitor, cyclosporin-A (1.0 *μ*mol/l), was used as a positive control.

### Statistics

Results were analyzed using an unpaired *t*-test, Mann–Whitney U test, a one-way analysis of variance followed by a Tukey post-test, or a two-way analysis of variance where appropriate. Statistical significance was achieved when *P*<0.05.

## Abbreviations

CsA: cyclosporin-A
DCF: CM-H2DCFDA
I/R: ischemia/reperfusion
MPTP: mitochondrial permeability transition pore
Mfn1: mitofusin 1
Mfn2: mitofusin 2
ROS: reactive oxygen species
SR: sarcoplasmic reticulum
