# Correction: Hearts deficient in both Mfn1 and Mfn2 are protected against acute myocardial infarction

**DOI:** 10.1038/s41419-021-03946-8

**Published:** 2021-06-30

**Authors:** A. R. Hall, N. Burke, R. K. Dongworth, S. B. Kalkhoran, A. Dyson, J. M. Vicencio, G. W. Dorn, D. M. Yellon, D. J. Hausenloy

**Affiliations:** 1 The Hatter Cardiovascular Institute, University College London, London, UK; 2 Division of Medicine, University College London, London, UK; 3 Center for Pharmacogenomics, Department of Internal Medicine, Washington University School of Medicine, St Louis, MO USA; 4 Cardiovascular and Metabolic Disorders Program, Duke-National University Singapore Graduate Medical School, Singapore, Singapore; 5 National Heart Research Institute Singapore, National Heart Centre Singapore, Singapore, Singapore

Correction to: *Cell Death and Disease* 10.1038/cddis.2016.139, published online 26 May 2016

The original version of this article unfortunately contained a mistake in an author name. The correct author name is GW Dorn 2^nd^. The original article has been corrected.

