# Supplementary figures and images for: Hearts deficient in both Mfn1 and Mfn2 are protected against acute myocardial infarction
Source: Cell Death Dis. 2016 May 26;7(5):e2238–. doi: 10.1038/cddis.2016.139 (PMC4917668; doi:10.1038/cddis.2016.139)

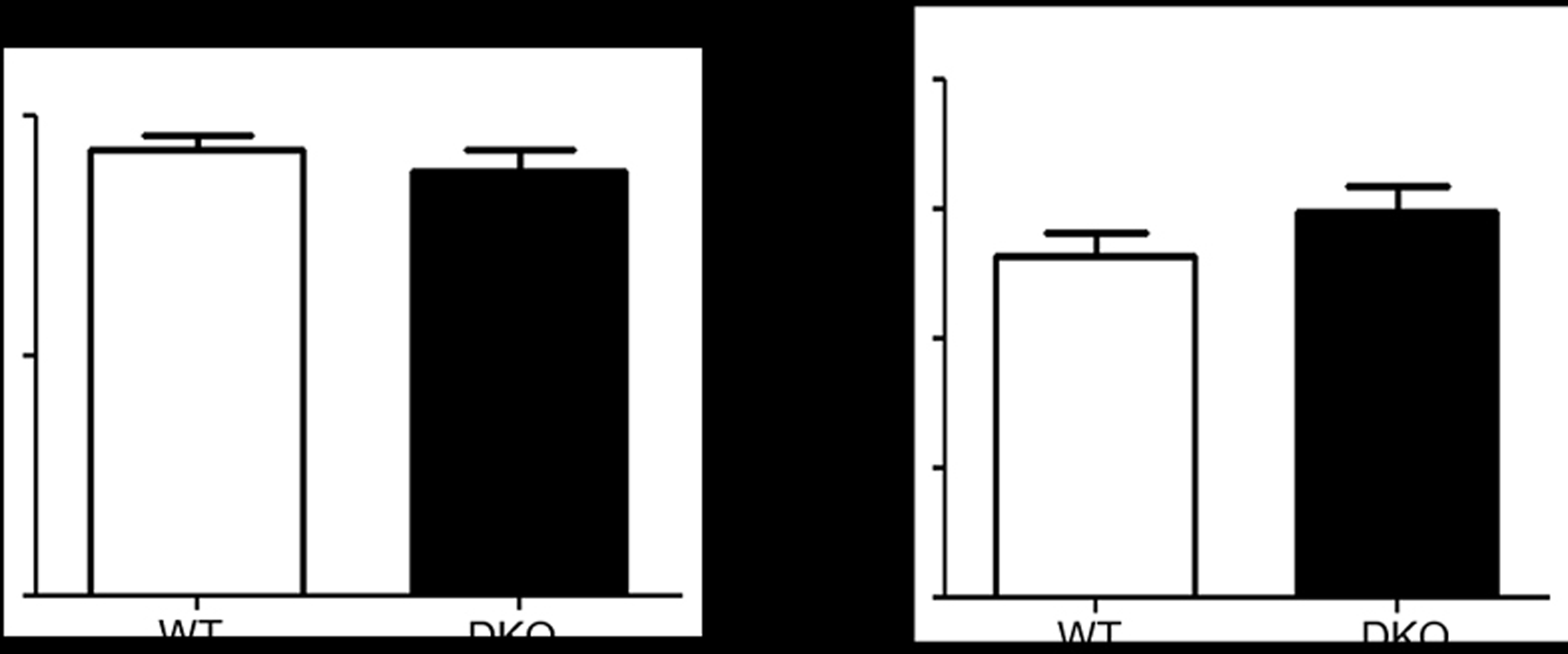

Supplement: Supplementary Figure 1 [file cddis2016139x1.tif]

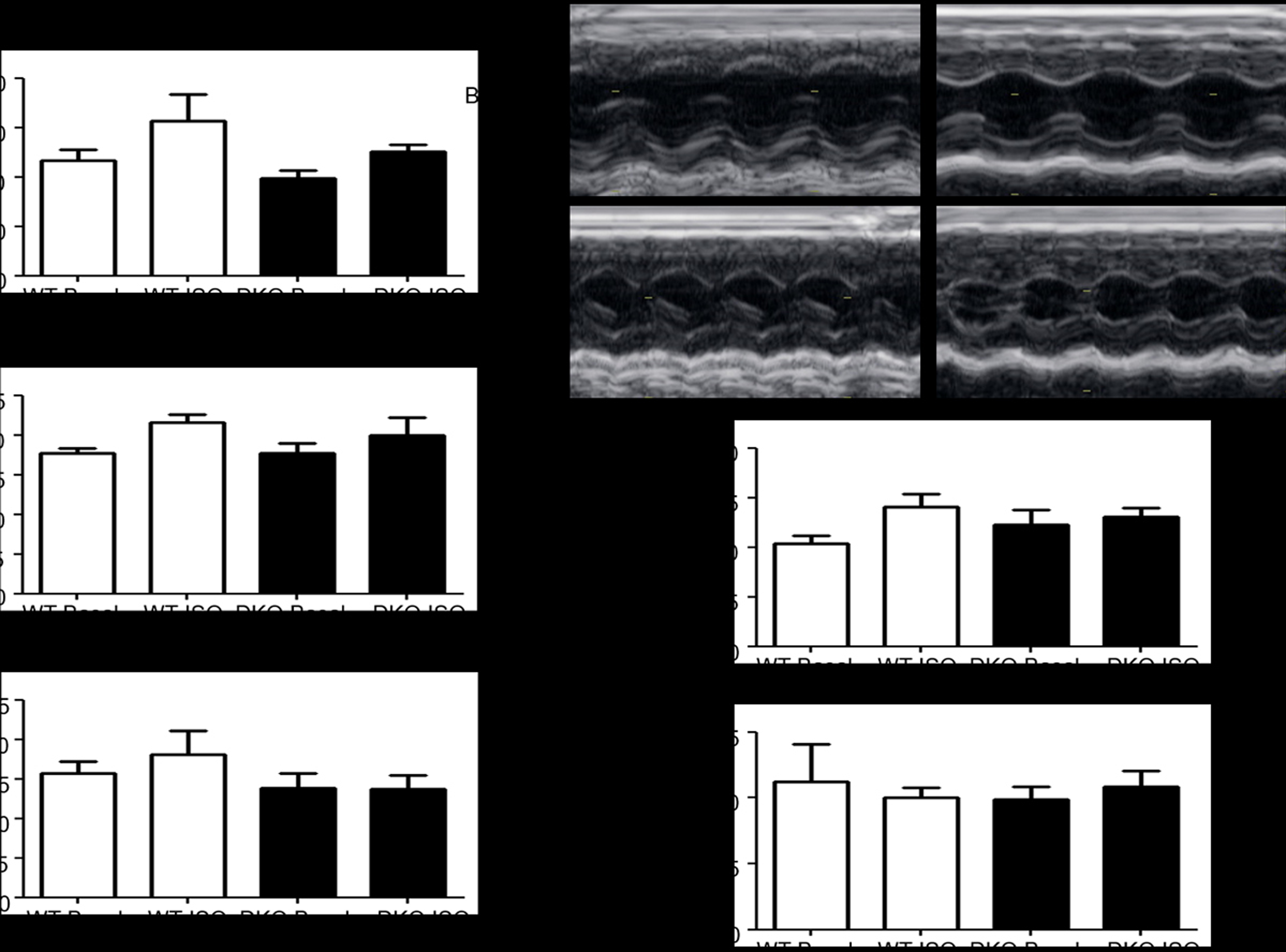

Supplement: Supplementary Figure 2 [file cddis2016139x2.tif]
