# Supplementary material for: Hearts deficient in both Mfn1 and Mfn2 are protected against acute myocardial infarction
Source: Cell Death Dis. 2016 May 26;7(5):e2238–. doi: 10.1038/cddis.2016.139 (PMC4917668; doi:10.1038/cddis.2016.139)
Supplement: Supplementary Figures [file cddis2016139x3.doc]

**Figure legends**

**Supplementary Figure 1**

**Effect of acute ablation of both cardiac Mfn1 and Mfn2 on heart weight and the area-at-risk.**

**(a)** There was no difference in heart/body weight between hearts deficient in both Mfn1 and Mfn2 (DKO) when compared to WT hearts. N=6/group. Error bars are SEM. Unpaired t-test. **(b)** There was no difference in area-at-risk (AAR) expressed as a percentage of the left ventricle (LV) volume between hearts deficient in both Mfn1 and Mfn2 (DKO) when compared to WT hearts. N=6/group. Error bars are SEM. Unpaired t-test.

**Supplementary Figure 2**

**Effect of acute ablation of both cardiac Mfn1 and Mfn2 on heart function and size.**

There were no significant differences between hearts deficient in both Mfn1 and Mfn2 (DKO) when compared to WT hearts with respect to:

**(a)** Fractional shortening at baseline and under Isoproterenol (ISO) stimulation.

**(c)** Left ventricular (LV) anterior wall thickness at end-systole at baseline and under ISO stimulation.

**(d)** LV anterior wall thickness at end-diastole at baseline and under ISO stimulation.

**(e)** LV posterior wall thickness at end-systole at baseline and under ISO stimulation.

**(f)** LV posterior wall thickness at end-diastole at baseline and under ISO stimulation.

**(b)** This figure shows a representative image of the left ventricle at end diastole and end systole at baseline and under ISO stimulation in hearts deficient in both Mfn1 and Mfn2 (DKO) and in WT hearts.

N=3/group. Statistical analysis was performed by a One-Way ANOVA with a Tukey post-test. Error bars are SEM.
